# Fuelled by creatine: exploring two copies of the creatine transporter SLC6A8 gene in rainbow trout

**DOI:** 10.1007/s10695-026-01723-y

**Published:** 2026-06-13

**Authors:** Andreas Borchel, Annika Müller-Eigner, Solvig Görs, Henrike Rebl, Alexander Rebl, Cornelia C. Metges, Tom Goldammer, Marieke Verleih

**Affiliations:** 1 Sea Lice Research Centre, Department of Biological Sciences, University of Bergen, Pb. 7803, Bergen, Norway; 2 Department of Anesthesiology and Perioperative, School of Medicine and Dentistry, University of Rochester, Rochester, NY USA; 3 Research Institute for Farm Animal Biology (FBN), Dummerstorf, Germany; 4 Institute of Cell Biology, Rostock University Medical Center, Rostock, Germany; 5 Faculty of Agriculture, Civil and Environmental Engineering, University of Rostock, Rostock, Germany

**Keywords:** Rainbow trout, Gene expression, Creatine transporter 1 CT1, SLC6A8, qPCR, Energy metabolism

## Abstract

The creatine transporter (SLC6A8 or CT1) is essential in creatine metabolism that serves as elementary cellular energy buffer. We identified the two *slc6a8* gene variants in the rainbow trout (*Oncorhynchus mykiss*) genome sharing 69.3% coding sequence similarity. Our analyses indicate that the gene duplication event occurred in a common ancestor of bony fishes, prior to the divergence of tetrapods. While one copy was lost in most vertebrate lineages, both are retained in teleosts. Expression analysis revealed tissue- and cell-specific regulation: both genes were highly expressed in the heart, s*lc6a8a* being predominantly expressed in most tissues and *slc6a8b* showing substantially elevated expression in muscle and salmonid embryonic CHSE-214 cells. Co-expression patterns with key creatine metabolism enzymes (*gatm*, *gamt*, *ckm*) support a fish-specific regulation of creatine homeostasis. Structural analysis confirmed the presence of 12 transmembrane domains and conserved functionally critical glycosylation motifs in both proteins. In addition, Slc6a8a contained an extracellular amino acid insertion with additional N-glycosylation motif. Fluorescent-labelled Slc6a8a and Slc6a8b proteins were localised to the plasma membrane in CHSE-214 cells, while high-performance liquid chromatography analysis confirmed creatine uptake. This study provides the first characterisation of the duplicated *slc6a8* genes in bony fish and suggests their sub-functionalisation during teleost evolution possibly reflecting functional specialisation of the paralogs. We hypothesise that one variant may primarily mediate creatine uptake, whereas the other could contribute to creatine release in distinct tissues under certain physiological conditions.

**Supplementary information:**

The online version contains supplementary material available at 10.1007/s10695-026-01723-y.

## Introduction

The creatine system is an important cellular energy buffer of high-energy phosphate bonds that rapidly replenish adenosine triphosphate (ATP) supply when needed. This is especially important in tissues with a high energy demand like brain, heart or muscle (Wyss and Kaddurah-Daouk 2000; Borchel et al. 2014). Due to the permanent loss of creatine/phosphocreatine by its spontaneous conversion to creatinine, creatine levels have to be continuously replaced. Creatine can be gained via nutrition or it can be synthesised intrinsically through a two-step mechanism involving the enzymes glycine amidinotransferase (GATM, alias AGAT) and guanidinoacetate N-methyltransferase (GAMT). The subsequent (de)phosphorylation is catalysed by creatine kinases (CKs) (Brosnan and Brosnan 2007). *GATM* encodes the first enzyme involved in creatine synthesis, which catalyses the rate-limiting step, *GAMT* encodes the second enzyme, which finally synthesises creatine. *CKM* encodes for the muscular creatine kinase, which is able to phosphorylate creatine using ATP. The creatine molecules subsequently have to be transported from the intestine or the organ of synthesis to the target organs. The cellular uptake of creatine from the bloodstream into cells is mediated by the sodium- and chloride-dependent creatine transporter 1 (SLC6A8 alias CT1), encoded by the gene *SLC6A8* (Sandoval et al. 1996; Borchel et al. 2014). To date, SLC6A8 is the only confirmed creatine importer in vertebrates (Jomura et al. 2022). It exhibits high affinity and specificity for creatine, whereas alternative transport pathways, such as vesicle-mediated uptake, remain unknown or insufficiently described (Bonilla et al. 2021). The transporter belongs to the solute carrier 6 (SLC6) transport family of secondary active Na^+^/Cl^−^ co-transporters, also referred to as neurotransmitter sodium symporters (NSS) (Kristensen et al. 2011). The NSS make use of the transmembrane sodium gradient to transport their wide range of substrates, like extracellular neurotransmitters, amino acids and osmolytes, across a biomembrane by the gated pore mechanism (Forrest et al. 2011; Pramod et al. 2013; Jayaraman et al. 2021). The SLC6 family includes 20 members in humans (including one pseudogene) and 16 members in teleost fish (Verri et al. 2012). The family is divided into four large subgroups on the basis of sequence similarity and substrate specificity: γ-aminobutyric acid (GABA) transporter, monoamine transporter, amino acid transporter I and amino acid transporter II/orphan transporter (Höglund et al. 2005; Bröer 2006). CT1 belongs to the first-mentioned subfamily of GABA transporter, together with the neurotransmitter transporter GAT1, GAT2 and GAT3; the betaine transporter BGT1; and the taurine transporter TauT (Bröer and Gether 2012).

In human, mutations in the CT1-encoding gene *SLC6A8* are linked to diseases like the creatine deficiency syndrome, mental retardation connected with developmental delay and intellectual disability (Salomons et al. 2001; Rosenberg et al. 2004; Santacruz and Jacobs 2016; Abdennadher et al. 2024). In mice, the importance of a functional creatine transport for immunity was proven. In murine macrophages, CT1-mediated creatine uptake influenced their polarisation and resulting phenotype (Ji et al. 2019). A loss of the transporter leads to disruption of the lymphocyte-mediated immune response in mice (Samborska et al. 2022). In aquatic vertebrates such as teleost fishes, however, the creatine system and the membrane transporter Slc6a8 remain poorly understood. Fish have developed specific metabolic strategies to adapt to the needs of different aquatic habitats. We have recently shown that teleost fish and mammals differ in regard to their creatine systems. While the two-step synthesis and the usage of creatine are spatially separated in mammals, they are not in fish. Unlike in mammals, the essential components of the creatine biosynthesis, *gatm* and *gamt*, are found highly expressed in muscle tissue from fishes (Borchel et al. 2014, 2019). Also, mammals and fish differ in the processing of the amino acids arginine and glycine, as well as in the metabolism of the GAMT co-substrate S-adenosylmethionine (SAM), all of which are essential for the first step of creatine biosynthesis. All together, these findings indicate the presence of a muscle-specific creatine synthesis in fish (Borchel et al. 2019).

The present study aimed to further enhance the knowledge on the teleost creatine system, particularly the transport. Hence, we characterised the duplicated rainbow trout (*Oncorhynchus mykiss*) *slc6a8* gene, encoding the creatine transporter Ct1. We investigated their specific structural and functional properties with regard to evolutionary conservation and their role as creatine transporters in a salmonid cell model, using various molecular techniques, including HPLC and qPCR analyses.

## Material and methods

### Real-time quantitative PCR (qPCR) expression analysis and RNA-seq data

We quantified the tissue-specific expression level of rainbow trout genes *slc6a8a* and *slc6a8b* in seven tissues (the heart, brain, spleen, head kidney, gills, muscle and liver) of unchallenged fish and their expression in the embryonic cell line CHSE-214 from Chinook salmon *Oncorhynchus tshawytscha* (Sigma-Aldrich) by qPCR using the SensiFast SYBR No-ROX Kit (Bioline, London, UK) on the LightCycler96 system (Roche). Tissue samples were used from a previous study (de los Ríos-Pérez et al. 2020). CHSE-214 cells were cultured as described previously (Rebl et al. 2019). Total RNA was extracted from approximately 5 mg of deep-frozen tissue and harvested CHSE-214 cells, homogenised in 1 mL TRIzol reagent (Invitrogen) and purified with the RNeasy Mini Kit (Qiagen). The concentration and purity of isolated RNA were evaluated using NanoDrop One (Thermo Fisher Scientific). Tissue- and cell-specific RNA was reverse-transcribed using the SensiFAST cDNA Synthesis Kit (Bioline/Meridian Bioscience), and 75 ng of cDNA was used per assay. The discriminating primers for *slc6a8a* or *slc6a8b*, listed in Table S2, were used to produce a 155-base pair (bp) or 174-bp fragment, respectively. The qPCR program included an initial activation step of 5 min at 95 °C, followed by 40 cycles of 15-s denaturation at 95 °C, 10-s annealing at 60 °C, 20-s elongation at 72 °C and final quantification for 10 s at 75 °C. Product size and quality of the resulting PCR products were confirmed using melting curve analysis and visualisation through separation in 2% agarose gels. Individual copy numbers were determined based on external gene-specific standard curves (10^7^–10^3^ copies per 5 μl; *R*^2^ > 0.999). Normalisation was performed with a factor based on the geometric mean of the expression of the suitable reference genes *eef1a1* (Bowers et al. 2008), *rps5* and *18 s* (Köbis et al. 2017).

The transcript level of the coding sequence for *gatm* (XM_036963960), *gamt* (XM_021603553) and *ckm* (XM_021617754) was determined using RNA-seq data from the BioProject PRJNA638521 analysing 16 heart and 16 skeletal muscle samples, from two different rainbow trout strains. Paired-end read data were mapped against an index fasta-file composed of coding-domain sequences of the genes of interest using bowtie2. Resulting sam-files were sorted and indexed using samtools. An overview of the number of mapped and unmapped reads per file was obtained using the BamIndexStats function of Picard-Tools. For each tissue of each fish the number of mapped reads per gene was divided by the total number of reads (in million reads) of this sample to account for different library sizes. Afterwards, a normalisation of the different genes was performed by dividing the obtained value by the sequence length (in kilobases) of each gene.

### Construction of *slc6a8*-expression plasmids and confocal microscopy

To determine the subcellular localisation of *slc6a8* gene variants from rainbow trout, we subcloned fragments spanning the open reading frame (ORF) of *slc6a8a* and *slc6a8b* as well as of the taurine transporter-encoding gene *slc6a6* via *Hin*dIII/*Mfe*I and *Eco*R1/*Afe*I into the vector pGEM-T Easy (Promega) using gene-specific oligonucleotides (Table S1). The gene coding for the taurine transporter Slc6a6 was chosen here as a comparative molecule for the localisation of the creatine transporter. The subsequent digestion was followed by the ‘in frame’ transfer into the mammalian expression vector v280 (Rebl et al. 2019) flagged with green fluorescent protein (GFP) or the purple fluorescent protein (mPlum). Both, GFP and mPlum, have been inserted previously into the v280 vector as described elsewhere (Sarais et al. 2020). All plasmid DNA was prepared endotoxin-free using the Endo-Free Plasmid Maxi Kit (Qiagen), and the correctness of all constructs was validated through sequencing.

The human embryonic kidney cell line 293 (HEK293; ATCC), cultured in EMEM medium (Sigma-Aldrich) as described previously (Rebl et al. 2019), was used to monitor the intracellular localisation of Slc6a8 from rainbow trout. HEK293 cells were transfected with 1 µg of either the constructed GFP-Slc6a8a and Plum-Slc6a8b or GFP-Slc6a8a and Plum-Slc6a6 fusion proteins using Lipofectamin 2000 (Thermo Fisher Scientific). Live-cell imaging was performed using the confocal microscope LSM 780 (Carl Zeiss Microscopy), equipped with a 63 × oil-immersion DIC objective and an incubation chamber to maintain ∼37 °C and 5% CO_2_ during the microscopic investigations. Images were acquired with a sampling pixel size of 0.066 × 0.066 µm. Hoechst 33342 dye (1 mg/mL, Sigma-Aldrich) was used to visualise the nuclei.

### Determination of creatine in salmonid and bovine muscle extracts and measuring of creatine uptake through SLC6A8 *in vitro* using HPLC

For the high-performance liquid chromatography (HPLC) analysis, skeletal muscle extracts from maraena whitefish (*Coregonus maraena*) were used due to the availability of pre-existing protein samples from an independent experiment (Brietzke et al. 2016). As both maraena whitefish and rainbow trout belong to the family *Salmonidae*, maraena whitefish samples were considered suitable for the intended comparative biochemical analysis. Bovine muscle samples were included as controls. No animals were sacrificed specifically for this study.

Muscle tissue of ~ 50 mg fresh weight was powdered finely in a mortar under liquid nitrogen and homogenised with a Precellys24 tissue homogeniser (Peqlab) in phosphate buffer (66 mM, pH 7.4, 4.5 times the tissue weight (Russell et al. 2014)). After three times freezing in liquid nitrogen and thawing at 37 °C for complete cell lysis, samples were centrifuged. The pellet was discarded while the supernatant was mixed with standard reaction media (Tris–HCL, 7.5 mM, pH 7.5; arginine, 150 µM; glycine, 75 µM; S-adenosylmethionine, 37 µM; dithiotheriol [DTT], 150 µM; and potassium phosphate buffer, 31.5 mM, pH 6.5). Samples were centrifuged for 10 min at 4 °C and 13,000 × g, and the supernatant was stored at − 20 °C until further processing. For subsequent determination of creatine in the muscle extracts of maraena whitefish and bovine (*n* = 4 each) by HPLC, according to Russell et al. (2014) with modifications, a 1200/1260 infinity Series system (Agilent Technologies) was used. Five µL of each sample were separated isocraticly on a 250 × 4 mm HyperClone ODS (C18) 120 Å column protected by a 4 × 3 mm C18 pre-column (both Phenomenex Inc) at constant flow of 1 mL/min and 25 °C using 10 mM Na_2_SO_4_ / 5 mM H_2_SO_4_ / 5 mM Na-1-hexane sulfonate as eluent. Creatin was detected by UV at 230 nm and quantified by multi-point calibration with external standards (0.25–5 mM).

Determination of the creatine concentration in CHSE-214 cells was carried out in 6-well plates (Greiner Bio-One, 60,000 cells per well). Cells were allowed to adhere for 24 h before either incubation with EMEM medium containing 20 mM of creatine monohydrate (Sigma-Aldrich) or left untreated for another 24 h. Subsequently, the cells were washed four times with ice-cold phosphate-buffered saline (PBS, Biochrom) and harvested as a pool of all six wells/plate in 150 µl lysis buffer (Promega). The cell suspension was centrifuged at 13,000 × g for 5 min at 4 °C, and the supernatant lysate was used for the subsequent profiling of the creatine concentrations using HPLC analysis. The experiment was repeated four times.

To detect the creatine uptake in pre-incubated CHSE-214 cells, the lysates were precipitated by 1.5 M HClO_4_, neutralised by 2 M K_2_CO_3_ (60:40:20 µL), centrifuged at 13,000 × g at 4 °C for 20 min and subsequently quantified by HPLC as described above. The quantification was performed by UV absorption at 210 nm to improve the sensitivity of the method and by 5-point calibration with external standards (0.1–2 mM).

### Phylogenetic analysis of SLC6 family

The sequence alignment and construction of the phylogenetic tree for the SLC6 family were conducted in MEGA 12 (Kumar et al. 2016). The evolutionary relationship was inferred using the maximum likelihood method and the Le and Gascuel (LG) amino acid substitution model (Le and Gascuel 2008) with gamma-distributed rate variation among sites. Node support was assessed by 1000 bootstrap replicates. Gaps and missing data were treated using partial deletion with a site coverage cutoff of 95%. Bootstrap values of more than 70% were assumed to be a reliable threshold for reliable grouping of proteins. Amino acid sequences used for the construction of the tree are listed in Table S2.

### *In silico* sequence analysis of *slc6a8* gene variants in rainbow trout

At the start of this study, the two gene entries XM_021566996 and XM_036947549 for *slc6a8* in rainbow trout were available in the NCBI (National Centre for Biotechnology Information; http://blast.ncbi.nlm.nih.gov/) database. To verify that both sequences represent independent gene variants of the creatine transporter, comparative sequence alignments were performed using the EMBOSS/Stretcher tool (https://www.ebi.ac.uk/Tools/psa/emboss_stretcher/). The InterProScan tool (https://www.ebi.ac.uk/interpro/search/sequence/) was used to identify functional protein domains/motives in the primary amino acid sequences of Slc6a8a (XP_036803444) and Slc6a8b (XP_021422671). Nucleotide sequence similarities were obtained using the EzBioCloud tool (https://www.ezbiocloud.net/tools/pairAlign (accessed March 2025) and protein blasts (NCBI-Blast-version 2.15.0). The Protter web application (https://wlab.ethz.ch/protter/start/, (Omasits et al. 2014)) was used to predict transmembrane domains as well as extra- and intracellular regions of the putative protein sequences. The NetNGlyc—1.0 server (Gupta and Brunak 2002) was used to predict N-glycosylation sites. We performed 3D-modelling using SWISS-MODEL (https://swissmodel.expasy.org/, Bienert et al. 2017) based on the cryo-EM structure of the human sodium- and chloride-dependent creatine transporter 1 (PDB entry 9KRH, chain A) as template. The quality of the generated models was assessed based on the GMQE and QMEANDisCo scoring functions. Visualisation of the resulting tertiary structure was done using UCSF Chimera (http://www.rbvi.ucsf.edu/chimera, Pettersen et al. 2004). Searches of the NCBI and Ensembl gene databases were performed to identify homologous sequences of *SLC6a8a* and *SLC6a8b* in teleost fishes and higher vertebrates (accessed September 2024). Synteny analyses were conducted using the Genomicus database (v98.01 http://genomicus.biologie.ens.fr/genomicus, Muffato et al. 2010). The sequences of rainbow trout *slc6a8a* (XM_036947549) and *slc6a8b* (XM_021566996) were provided as BLAST queries in the Genomicus database to identify the arrangement of synteny blocks for the corresponding orthologous genes in the genomes of other teleosts and higher vertebrates.

## Results and discussion

### The creatine transporter gene *slc6a8* is duplicated in rainbow trout

A search in the gene database of the National Centre for Biotechnology Information (NCBI) identified two entries for the creatine transporter Slc6a8-encoding gene on chromosome 16 (NC_048580.1) from rainbow trout: (i) XM_036947549 (bases 64,446,798..64,485,638, complement) and (ii) XM_021566996 (bases 66,738,326..66,767,036, complement). Subsequently, we termed them as *slc6a8a* and *slc6a8b*. The duplicated *slc6a8* genes differ in their exon–intron structure. While *slc6a8a* comprises 14 exons and 13 introns, *slc6a8b* is composed of 13 exons and 12 introns. *Slc6a8a* displays an additional intron in the coding region, located 28 base pairs (bp) downstream of the start codon. Rainbow trout *slc6a8a* and *slc6a8b* open reading frames (ORF) comprise 2055 and 1974 bp, respectively. Both ORFs share 69.3% sequence similarity. The encoded proteins expand 684 amino acids (aa) for SLC6A8a (XP_036803444) and 657 aa for SLC6A8b (XP_021422671). Both sequences share 64.5% identical, 12.3% strongly similar and 7.9% weakly similar aa.

**Fig. 1:**
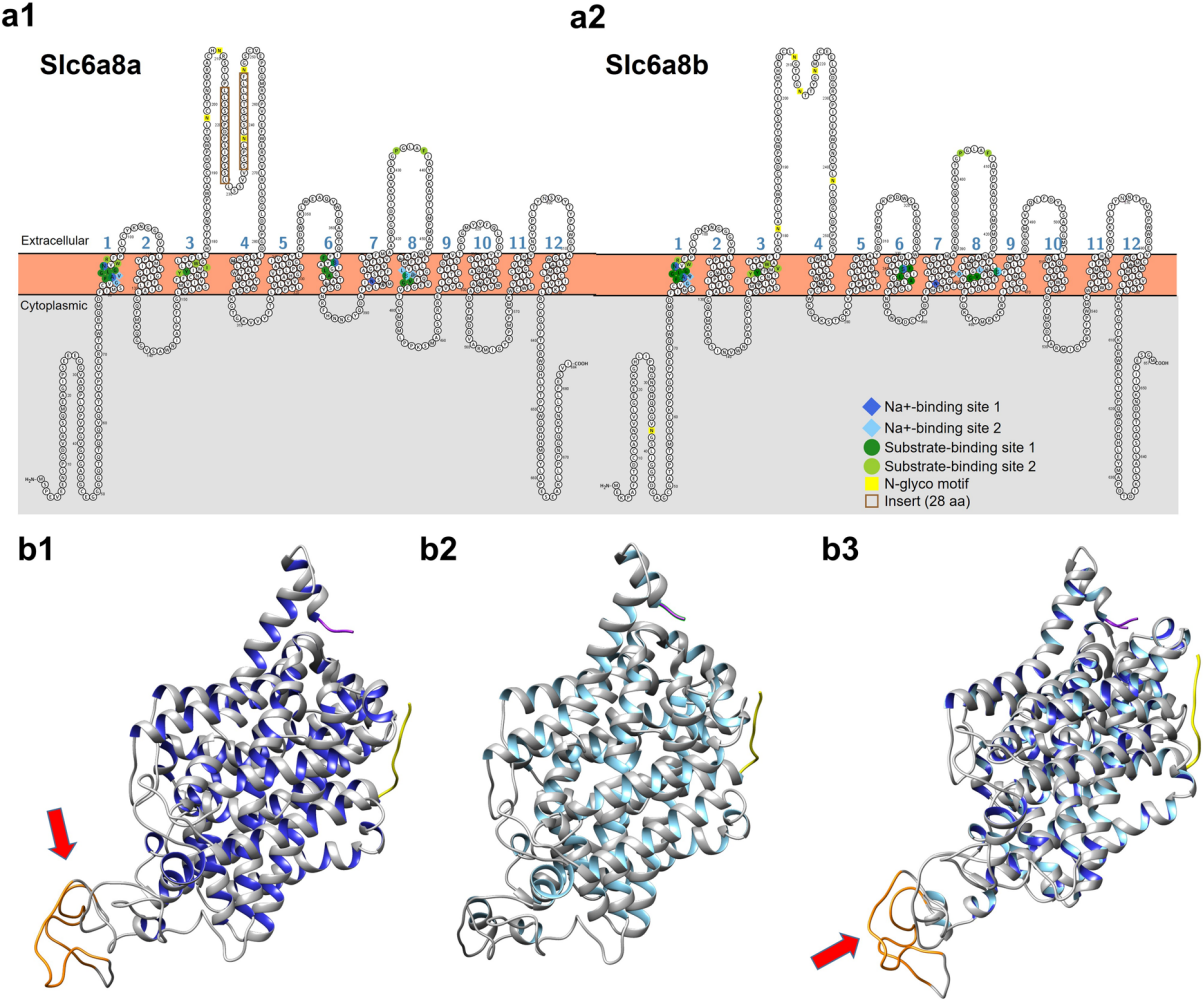
Protein structure of Slc6a8 variants from rainbow trout. (**a**) Predicted membrane topology for (**a1**) Slc6a8a and (**a2**) Slc6a8b from rainbow trout generated by Protter. Selected residues are colour-coded according to in the legend. The brown boxes frame a 28 aa insert of variant Slc6a8a when compared to Slc6a8b. (**b**) Tertiary structure of (**b1**) Slc6a8a (grey/dark blue), (**b2**) Slc6a8b (grey/turquoise) and (**b3**) merged for Slc6a8a and Slc6a8b. The N- and C-termini are indicated by yellow and purple colour, respectively. The insert of variant Slc6a8a (starting at position 214) is highlighted in orange and indicated by a red arrow

The 28 additional aa of Slc6a8a are located within a two-part insert comprising two 14-nucleotide segments at nucleotide positions 215–228 and 234–247 (Fig. S1). It is predicted to be part of a large extracellular loop located between transmembrane domains (TM) 3 and 4 (Fig. 1 (a1, a2)). The insert adds an additional N-glycosylation motif at residue N238, between the well-defined N-glycosylation sites at residues N192 and N197 within the same loop, which have been shown to be functionally critical using glycosylation-deficient mutants of the rat creatine transporter (Straumann et al. 2005). Apart from that, the predicted membrane topology of Slc6a8a and Slc6a8b in rainbow trout is highly similar: consistent with published findings about the structure of transporters belonging to the SLC6 family (Santacruz and Jacobs 2016), both proteins contain 12 TMs, and their N- and C-termini are facing the cytoplasmic side of the membrane (Fig. 1 (a1, a2)). Despite localisation in the membrane, no signal peptide but 4 or 6 N-glycosylation motifs were predicted for Slc6a8a or Slc6a8b, respectively. Moreover, sodium binding sites were found in TM 1, 3, 6, 7 and 8 as well as substrate binding sites in TM 1, 3, 6 and 8. Based on findings in the prokaryotic leucine transporter LeuT (Yamashita et al. 2005), the substrate binding sites contribute to the permeation pathway of the gate-pore mechanism of substrate transport, with TM 1 and 6 in the middle forming the centre for the co-transport of Na^+^/Cl^−^ and the substrate (Yamashita et al. 2005; Bröer and Gether 2012; Colas et al. 2020).

The size and sequence similarity of the different transmembrane domains were similar between Slc6a8a and Slc6a8b, with identical sections for the first, the sixth and the eighth transmembrane domains (Fig. S1). However, the sequence identity of the intracellular domains at the N- and C-termini was much lower (about 28 and 40% identical aa, respectively). These structural characteristics were also confirmed by a three-dimensional modelling of the protein structures (Fig. 1 (b1–b3)). The generated models of rainbow trout Slc6a8a and Slc6a8b showed good overall quality, with a GMQE score of 0.70 or 0.77 as well as a QMEANDisCo global score of 0.78 ± 0.05 or 0.80 ± 0.05, respectively. Both proteins exhibit a high degree of positional conservation in their predicted α-helical regions, with all major transmembrane helices being largely identical in sequence and length (Fig. 1 (b3)). The extracellular loops, which connect these helices, were superposable; only the insert of Slc6a8a formed an additional loop. In contrast, neither the N-termini nor the C-termini of both proteins showed significant similarity.

### Duplicated copies of *slc6a8* are retained in the majority of teleost fish species, whereas one gene variant was lost during evolution

A search of the NCBI and Ensembl gene databases identified homologous sequences of *slc6a8a* and *slc6a8b*, comprising orthologues in 12 members of the *Salmonidae* family (Table 1). As in rainbow trout, we found two *slc6a8* gene copies in 11 of the 12 salmonid species, though not for Arctic char. Orthologues of both gene variants were detected in 85% of the teleost fish species with database entries. In contrast, all identified orthologues in birds can be assigned to *Slc6a8a*, while those in reptiles and amphibians represent to 99% gene variant *slc6a8b*. The only exceptions are the database entries for one crocodile (XM_019536775) and two alligator species (XM_006278196 and XM_025210762), which are assigned to *slc6a8a* or not clearly to neither of the two variants, respectively. Furthermore, both gene variants (XM_012960512 and XM_002935493) are present in the amphibian western clawed frog *Xenopus tropicalis* (Table 1). Interestingly, all GeneBank entries for *SLC6A8* from mammals represent gene variant *SLC6a8b*. The findings indicate that the duplication event has its evolutionary origin in a common ancestor of bony fishes and before the split of the tetrapods ~ 380 million years ago (mya, Clack 2009), presumably due to a local gene duplication event. In the further course of evolution, the presence of a single copy of the creatine transporter appears to be functionally sufficient in tetrapods, as one of the gene variants was lost in amphibians, reptiles, birds and mammals. Both variants must have been preserved throughout tetrapod and amniote evolution until the emergence of mammals ~ 320 million years ago (Kumar et al. 2022). Interestingly, variant *SLC6A8a* was preserved in birds, whereas in reptiles and the majority of other tetrapods, it is variant *SLC6A8b*. However, why birds have retained *SLC6A8a* in contrast to the other groups remains purely speculative and requires further investigation of creatine transport in these taxa.

**Table 1:** *SLC6A8* sequences identified in rainbow trout, other salmonids and selected higher vertebrates

	NCBI gene symbol	NCBI/Ensembl gene ID	NCBI /Ensembl nucleotide accession number	Chromosome	mRNA length (nt)	NCBI/Ensembl protein accession numbe**r**	Protein length (aa)	Protein identity (%)^a^
SLC6A8a								
*Salmonidae*								
*Oncorhynchus mykiss*	si:ch211-117c9.5	110492540	XM_036947549	16	3436	XP_036803444	684	100
*Oncorhynchus kisutch*	si:ch211-117c9.5	109880037	XM_031803489	LG24	4691	XP_031659349	685	99.1
*Oncorhynchus keta*	si:ch211-117c9.5	118371140	XM_052481835	27	4439	XP_052337795	684	99.9
*Oncorhynchus* gorbuscha	si:ch211-117c9.5	124045587	XM_046364976	LG10	2565	XP_046220932	684	99.7
*Oncorhynchus nerka*	si:ch211-117c9.5	115101991	XM_065005271	LG20	4339	XP_064861343	684	99.9
*Oncorhynchus tshawytscha*	si:ch211-117c9.5	112222304	XM_042304094	LG22	3688	XP_042160028^b^	615	87.7
*Coregonus* *clupeaformis*	si:ch211-117c9.5	121546097	XM_041857117	30	3028	XP_041713051	684	98.8
*Salmo trutta*	si:ch211-117c9.5	115168533	XM_029723911	30	3701	XP_029579771	684	99.6
*Salmo salar*	si:ch211-117c9.5	106566567	XM_045693005	ssa13	3918	XP_045548961	684	99.6
*Salvelinus alpinus*	Not identified							
*Salvelinus fontinalis*	si:ch211-117c9.5	129861114	XM_055932254	8	4380	XP_055788229	684	99.4
*Salvelinus namaycush*	si:ch211-117c9.5	120060786	XM_039010209	16	2842	XP_038866137^b^	608	88.3
*Hucho hucho*	si:ch211-117c9.5	ENSHHUG00000017384	ENSHHUT00000028577	Unknown	2763	ENSHHUP00000027483	685	98.1
*Selected higher vertebrates*								
*Takifugo rubripes*	si:ch211-117c9.5	101072610	XM_003963293	3	3619	XP_003963342	667	79.1
*Danio rerio*	si:ch211-117c9.5	100150452	XM_001922928	23	2740	XP_001922963	652	75.5
*Xenopus tropicalis*	SLC6A8l	100127575	XM_012960512	4	7030	XP_012815966	654	71.0
*Gallus gallus*	SLC6A8	100861584	XM_015293329	12	2540	XP_015148815	647	71.5
*Mus musculus*	Not identified							
*Homo sapiens*	Not identified							
SLC6A8b								
*Salmonidae*								
*Oncorhynchus mykiss*	SLC6A8	110492584	XM_021566996	16	6433	XP_021422671	657	100
*Oncorhynchus kisutch*	SLC6A8	109869389	XM_020459503	LG24	6469	XP_020315092	657	100
*Oncorhynchus keta*	SLC6A8	118359555	XM_035738061	27	6412	XP_035593954	657	100
*Oncorhynchus* gorbuscha	SLC6A8	124045550	XM_046364911	LG10	2909	XP_046220867	657	100
*Oncorhynchus nerka*	SLC6A8	115101964	XM_029621423	LG20	5766	XP_029477283	657	100
*Oncorhynchus tshawytscha*	SLC6A8	112222349	XM_024385139	LG22	6450	XP_024240907	657	100
Coregonus *clupeaformis*	SLC6A8	121546059	XM_041857065	30	3886	XP_041712999	656	98.8
*Salmo trutta*	SLC6A8	115168574	XM_029723975	30	6381	XP_029579835	657	99.9
*Salmo salar*	SLC6A8	106566494	XM_014134576	ssa13	6432	XP_013990051	657	99.9
*Salvelinus alpinus*	SLC6A8	112069770	XM_024137146	Unknown	6974	XP_023992914	657	99.6
*Salvelinus fontinalis*	SLC6A8	129861192	XM_055932391	8	6490	XP_055788366	657	99.6
*Salvelinus namaycush*	SLC6A8	120061417	XM_039011211	16	6435	XP_038867139	657	99.6
*Hucho hucho*	SLC6A8	ENSHHUG00000005394	ENSHHUT00000009409	Unknown	2633	ENSHHUP00000009129	657	99.7
*Selected higher vertebrates*								
*Takifugo rubripes*	SLC6A8	101073125	XM_029834244	3	7925	XP_029690104	663	81.7
*Danio rerio*	SLC6A8	567544	NM_001327868	8	2223	NP_001314797	652	86.5
*Xenopus tropicalis*	SLC6A8	100489909	XM_002935493	8	7984	XP_002935539	644	73.2
*Gallus gallus*	Not identified							
*Mus musculus*	SLC6A8	102857	NM_133987	X	3992	NP_598748	640	72.1
*Homo sapiens*	SLC6A8	6535	NM_005629	X	6931	NP_005620	635	72.5

^a^In relation to respective rainbow trout orthologue

^b^Partial sequence with shortened C-terminus

This divergence in gene retention, whereby multiple functional gene copies are retained in teleost fish while being lost in higher vertebrates, is a recurring phenomenon. The genomes of true bony fishes, and salmonids in particular, generally contain a large number of genes which are present in at least two copies (Robertson et al. 2017; Parey et al. 2022), due to frequent independent duplications (Robinson-Rechavi et al. 2001) and whole-genome duplication (WGD) events (Hurley et al. 2007; Macqueen and Johnston 2014). Nevertheless, the fate of most duplicated genes is eventual loss or degradation to pseudogenes (Lynch and Conery 2000). The selective retention of multiple gene copies in teleost fish, along with associated sub- or neofunctionalization, contributes to phenotypic complexity and the subsequent occupation and exploitation of niche habitats in the aquatic environment. It is assumed that despite evolutionary gene loss and the constant process of rediploidization, the retention of duplicated genes, e.g. in the zebrafish genome, can be up to 20% (Postlethwait et al. 2004; Woods et al. 2005). Gene duplication is thought to be one major driver of speciation and species richness in the group of bony fish (Meyer and Schartl 1999; Taylor et al. 2001).

**Fig. 2:**
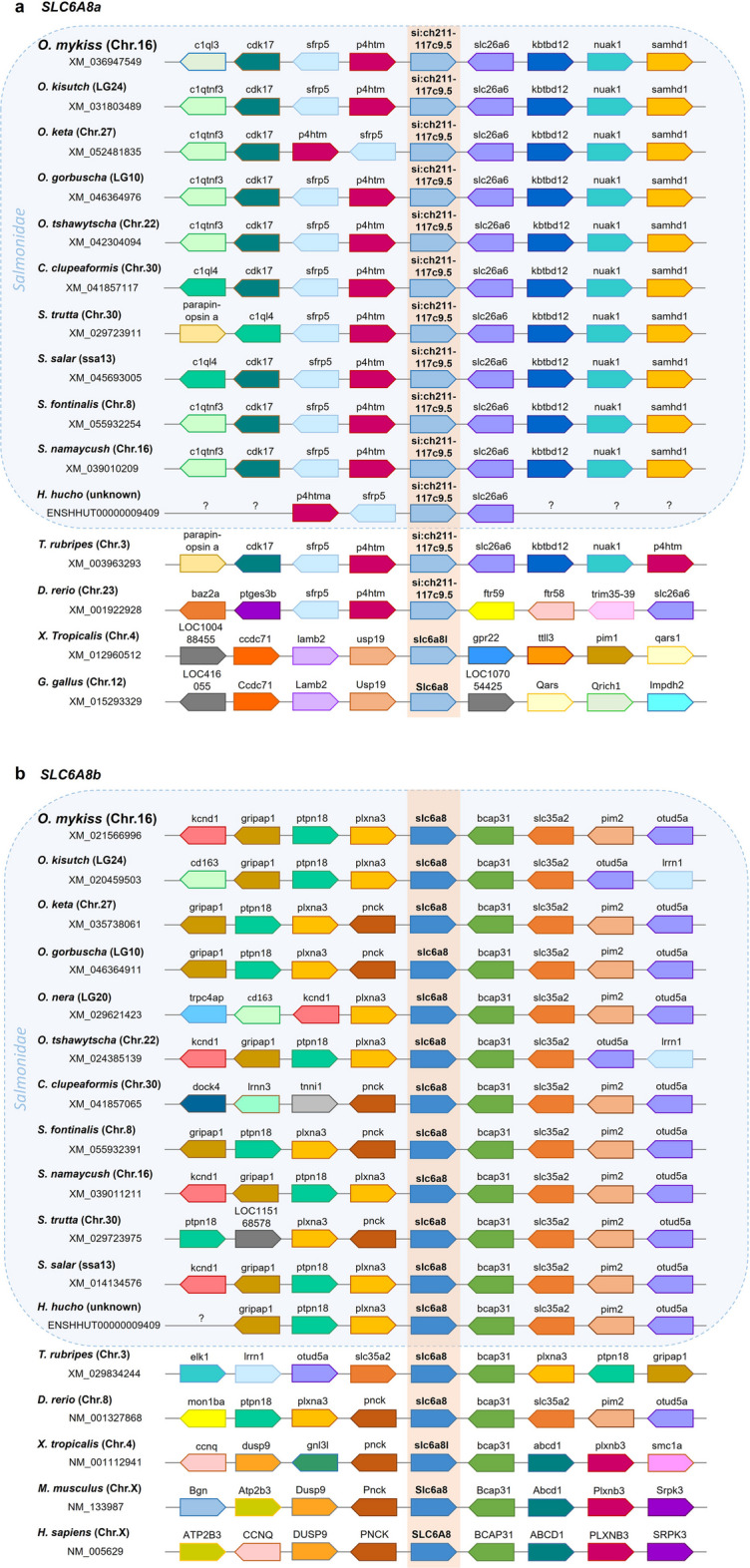
Comparative synteny analysis of *SLC6A8* genes. Shown are the adjacent upstream and downstream genes of **a** *SLC6A8a* (*si:ch211-117c9.5*) and **b** *SLC6A8b* from rainbow trout and other vertebrates including all orthologues from salmonid fishes (shaded blue boxes) using the Genomicus, NCBI and Ensembl genome databases. The NCBI nucleotide accession codes, species names and chromosomal location are listed on the left side. The bar lengths are not proportional to the real distances between genes on the chromosome. The arrows indicate the transcriptional direction on the respective chromosome. Same colours reflect the same gene across species. Note that the assembly of the salmonid huchen (*H. hucho*) is only on scaffold level and information on the flanking genes is therefore limited

A synteny analysis allowed us to further deduce the ancestry of the *slc6a8* genes (Fig. 2). Both gene variants are flanked by a different set of genes, suggesting that *slc6a8a* and *slc6a8b* are paralog. In detail, gene variant *slc6a8a* of salmonids is surrounded by a downstream gene group comprising *slc26a6*, *kbtbd12*, *nuak1* and *samhd1* (Fig. 2a). In contrast, only *slc26a6* from this group belongs to the immediate downstream genes in Japanese pufferfish *Takifugu rubripes* and zebrafish *Danio rerio*. All *slc6a8a* orthologues from teleosts are moreover located in close upstream proximity to *p4htm* and *sfrp5*. The representative orthologous genes from amphibian (*X. tropicalis*) and bird (chicken *Gallus gallus*) share none of the flanking genes identified in teleost fishes, but they have the upstream gene group of *USP19*, *LAMB2* and *CCDC71*, as well as the downstream gene *QARS1*, in common (Fig. 2a). *SLC6A8b* orthologues are consistently flanked by *bcap31* (Fig. 2b). Furthermore, *slc6a8b* genes from teleost fish are closely located to *plxna3* (except for *C. clupeaformis*), *slc35a2*, *pim2* (except for *O. kisutch, O. tshawytscha* and *T. rubripes*) and *otud5*/*otud5a*. The direct proximity to *ptpn18*, *gripap1* and *pnck* is also common to the majority of the salmonid *slc6a8b* genes. In *T. rubripes*, these flanking genes are located contrary to their position upstream or downstream of *slc6a8b* in the other teleosts, presumably due to a reverse insertion after chromosomal strand break or translocation (Kai et al. 2005). The gene variant *SLC6A8b* in higher vertebrates, including *X. tropicalis*, the house mouse *Mus musculus* and the human *Homo sapiens*, shares conserved upstream (*DUSP9*, CCNQ and *ATP2B3*) and downstream (*ABCD1*, *PLXB3* and *SRPK3*) gene groups.

**Fig. 3:**
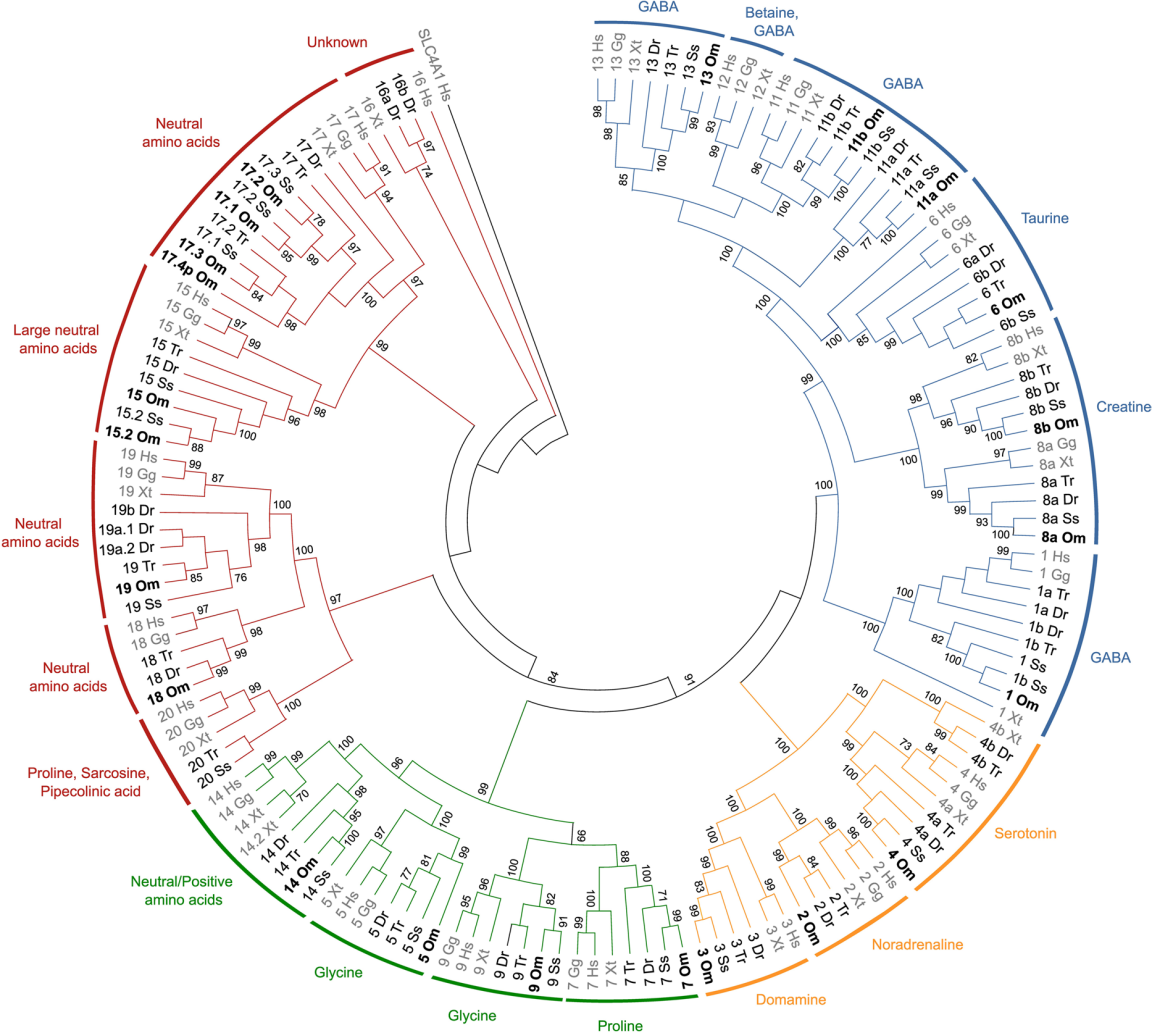
Phylogenetic analysis of SLC6 transporter family. The tree was constructed with MEGA12 using the maximum likelihood method and the Le and Gascuel (LG) amino acid substitution model with gamma-distributed rate variation among sites (five discrete categories). Node support was assessed by 1000 bootstrap replicates. Labels shown in black indicate selected amino acid sequences from teleost fishes (Om, *Oncorhynchus mykiss*; Ss, *Salmo salar*; Tr, *Takifugu rubripes*; Dr, *Danio rerio*), those from rainbow trout are highlighted in bold letters. Selected sequences from higher vertebrates (Xt, *Xenopus tropicalis*; Gg, *Gallus gallus*; Hs, *Homo sapiens*) are indicated by grey letters. The full list of used protein sequences is provided in Table S1. The four SLC6 subfamilies are highlighted in blue (GABA transporter), orange (monoamine transporter), green (amino acid transporter I) and red (amino acid transporter II/orphan) branches and labels. Anion exchanger SLC4A1 from human was used as outgroup. Only bootstrap values above 70% are shown. No fish-specific sequences were available for betaine transporter SLC6A12. The encoding gene for the SLC6A10 transporter is listed as a pseudogene and the family members have therefore been omitted from the tree

The relationship between the encoded rainbow trout paralogs Slc6a8a and Slc6a8b and their orthologs in other species and within the SLC6 protein family was further investigated using protein BLAST analysis. The results revealed amino acid sequence similarities of over 87% for Slc6a8a and more than 98% for Slc6a8b from rainbow trout when compared to all identified complete protein sequences of other salmonids (Table 1). They are furthermore between 71.0 and 79.1% (SLC6A8a) or 72.1 and 86.5% (SLC6A8b) identical to orthologues analysed from higher vertebrates. Furthermore, we constructed a phylogenetic tree of the 19 members of the SLC6 family of membrane transporters, including all known protein sequences from four teleost species (*O. mykiss*, *S. salar*, *T. rubripes* and *D. rerio*) and three species of higher vertebrates (*X. tropicalis*, *G. gallus* and *H. sapiens*; Fig. 3). Overall, members of the SLC6 protein family clustered into four main branches based on their substrate: amino acid transporters I, amino acid transporters II, monoamine transporters and GABA transporters. Piscine proteins are clearly separated from orthologues of higher vertebrates and build distinct clades with 65.0 to 100% bootstrap support. The sequences of the 16 known rainbow trout SLC6 family members (Slc6a1-9, Slc6a11, Slc6a13-15, Slc6a17-19) were sub-clustered into clades with orthologous proteins from the specific family members of the other species. Within these, the rainbow trout Slc6a8 paralogs Slc6a8a and Slc6a8b group together with identified orthologous variants (compare Table 1, Fig. 3).

The duplication rate among the SLC6 family members is remarkably high, with nearly 50%. Duplicated proteins were identified for 9 of the 19 membrane transporters of the SLC6 family in teleost fish. Even four paralogue sequences of Slc6a17 were found in rainbow trout (encoding genes located on chromosome 7, 9, 16 and 17) and three in Atlantic salmon (chromosome ssa12, ssa13 and ss15; Table S1). The fact that slightly half of the members of the SLC6 family are preserved in several gene copies indicates the functional importance and individual specialisation of these variants. Schneider and colleagues reported reduced selection pressure on *slc6a8* in salmonid fishes due to a combined way of relaxed and diversifying selection (Schneider et al. 2019). Relaxed selection pressure on one of the duplicated gene copies, with continued strong selection on the other, can lead to silencing or neofunctionalisation (Roth et al. 2007; Lahti et al. 2009). To our knowledge, no published study on the SLC6-family in teleost fish has focused on the functional analysis of the identified gene duplicates. Reports on teleost fish that explore the evolutionary history or the ontogenetic and stress-specific expression pattern of *slc6a8* in *D. rerio* (Wang et al. 2007, 2010), olive flounder *Paralichthys olivaceus* (Zou et al. 2024) and medaka *Oryzias latipes* (Kinjo et al. 2013) so far did not consider creatine transporter gene variants. Studies focusing on other SLC families, though, show specialised functions of duplicated members for *slc15a1* in killifish mummichog *Fundulus heteroclitus* (Bucking and Schulte 2012) and for *slc34a2* transporters in salmonid fish (Verri and Werner 2018).

### *Slc6a8a* and *slc6a8b* genes show variant-specific expression patterns

**Fig. 4:**
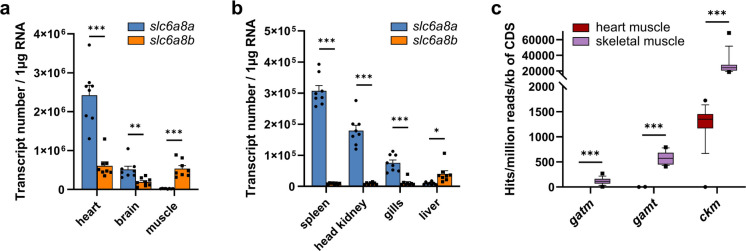
Tissue-specific expression profile of *slc6a8* gene variants in rainbow trout. Bars represent mean high **(a)** or moderate to low **(b)** transcript levels (*n* = 8) of *slc6a8a* (blue bars) and *slc6a8b* (orange bars), determined in the heart, brain, spleen, head kidney, gills, muscle and liver. Copy numbers were normalised to three reference genes; error bars represent SEM. (**c)** Expression of creatine system genes *gatm*, *gamt* and *ckm* in heart (dark red box-plot) and skeletal muscle (purple box-plot) of rainbow trout, determined by analysing RNA-seq data. Upper and lower edges mark the upper and lower quartiles, the lines in the boxes the medians, the whiskers the 10th and 90th percentiles; dots represent outliers. Error bars represent ± SEM. Asterisks indicate statistical significance: *p < 0.05; **p < 0.01; ***p < 0.001

To explore the potential functional differences among the *slc6a8* gene variants, we profiled their expression patterns across seven distinct tissues in untreated adult rainbow trout. The transcription level of gene variants *slc6a8a* and *slc6a8b* peaked in the heart, compared to the other tissues and differed significantly between the two variants (Fig. 4a, b). Overall, *slc6a8a* was also highly expressed in brain, spleen and head kidney; moderately expressed in gill tissue; and negligible in muscle and liver tissue. At first glance, the comparatively lower transcript abundance observed in gill tissue may appear inconsistent with the high energetic demands of branchial ionocytes associated with active ion transport. However, this pattern may reflect the cellular heterogeneity of the branchial epithelium, as whole-tissue qPCR measurements do not resolve expression at the level of specific cell populations such as ionocytes (Dymowska et al. 2012). Although ionocytes are characterised by high energetic demands associated with ion transport, branchial ATP homeostasis may additionally rely on localised glycolytic and mitochondrial ATP production (Tseng and Hwang 2008). Therefore, the present data do not allow conclusions regarding the relative contribution of creatine-dependent energy buffering mechanisms within specific gill cell types. In contrast, *slc6a8b* was strongly expressed in the muscle, moderately in brain, weakly in the liver, and negligible in the remaining tissues. While the transcript level of *slc6a8b* in muscle tissue clearly and significantly exceeded that of *slc6a8a* by 25-fold, *slc6a8a* was predominantly expressed in most of the other tissues.

In general, our expression data are consistent with the few known results obtained from studies in zebrafish, olive flounder and largemouth bass *Micropterus salmoides* (Wang et al. 2007, 2010; Zou et al. 2024; Yu et al. 2025), albeit previous studies focused on only one variant of the *slc6a8* gene (*slc6a8b*), despite the availability of multiple sequences in public databases. Functional studies on duplicated genes of the SLC6 transporter family in teleosts are lacking. However, reports on other duplicated members of the SLC family suggest that the retention of paralogs is often associated with functional specialisation. Verri and Werner (2018) compiled findings on the duplication of type II Na^+^-phosphate cotransporter family members slc34a1 (*slc34a1a* and *slc34a1b*) and slc34a2 (*slc34a2a* and *slc34a2b*; Verri and Werner 2018) and reported divergent substrate affinities as well as tissue-specific expression patterns. A study on two *slc15a1* genes in killifish mummichog identified their specific expression in relation to water salinities (Bucking and Schulte 2012). It can be hypothesised that the tissue-specific expression of *slc6a8a* and *slc6a8b* shown here is also based on specialisation.

The expression analysis also confirmed differences in the expression of *SLC6A8* in mammals. As shown recently, mammals hardly synthesise creatine in their muscles, whereas muscular synthesis appears to be pronounced in fish (Borchel et al. 2019). The specific expression of *slc6a8a* and *slc6a8b* in the heart and muscle of rainbow trout (Fig. 4a) prompted us to further investigate the general differences in the creatine synthesis of these organs. To this end, we determined the expression levels of *gatm*, *gamt* and *ckm*, encoding remaining central enzymes of the creatine system, using RNA-seq data from rainbow trout. *Gatm* and *gamt* were expressed in the skeletal muscle (118 and 575 hits per million reads per kilobase [RKB], each), but only to very low extent in heart muscle (< 2 hits/per million RKB, Fig. 4c). In contrast, *ckm* was expressed in both tissue types, with mean read counts of 1.2 and 28 k hits/million RKB. Expression levels were 20-fold higher in skeletal than in heart muscle. These findings suggest distinct creatine metabolism between both tissues in rainbow trout. Unlike skeletal muscle, the heart appears unable to synthesise its own creatine. As it still requires creatine, as shown by a strong expression of *ckm*, heart muscle is likely dependent on creatine import. Skeletal muscle, by contrast, shows even higher transcript levels of *ckm* and may require more creatine but appears capable of synthesising it endogenously. This may reduce the needs for creatine-import or may, under certain physiological conditions, even favour creatin export. Thus, it seems plausible to utilise two different Slc6a8 transporter variants in heart and skeletal muscle. The paralogs may differ in their biochemical properties, such as substrate affinity. It is also conceivable that both transporters operate in opposite directions in rainbow trout, with Slc6a8a functioning as an importer and Slc6a8b as an exporter.

However, as members of the SLC6 family are secondary active transporters whose transport direction is determined by the electrochemical gradients of coupled ions, particularly Na^+^ and Cl^−^, rather than by transporter identity alone (Guimbal and Kilimann 1993; Peral et al. 2010), such a scenario would require specific thermodynamic conditions. In particular, net creatine export via Slc6a8b would likely only occur if intracellular creatine concentrations are sufficiently elevated and/or local Na^+^ gradients are reduced adequately to diminish the inward driving force typically favouring creatine uptake into the cell. Such conditions may occur in skeletal muscle with high endogenous creatine synthesis and intense metabolic activity, where fluctuations in ion homeostasis and membrane potential have been described during sustained contraction and metabolic stress (Wyss and Kaddurah-Daouk 2000; Wallimann et al. 2011). Nevertheless, this interpretation remains speculative at present. Consequently, the proposed differential directionality of the two paralogs requires experimental verification, including functional transport studies addressing ion dependence and transport energetics.

### Spatial arrangement of Slc6a8 in cellular substructures

**Fig. 5:**
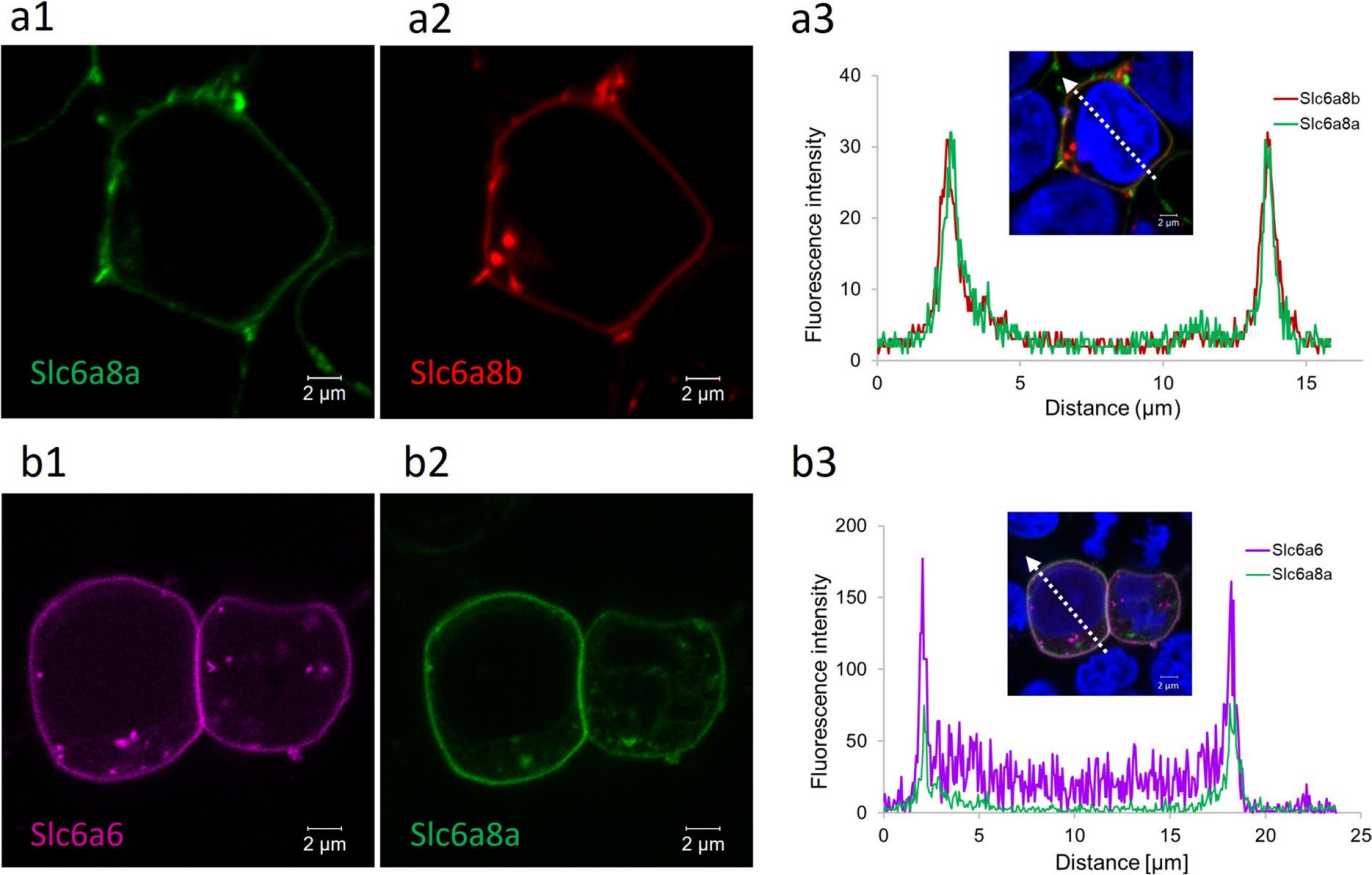
Cellular localization of Slc6a8 variants. Confocal analysis of HEK-293 cells transfected with GFP-Slc6a8a (**a1**, **b2**; green), Plum-Slc6a8b (**a2**; red) or reference Plum-Slc6a6 (**b1**; purple). HEK-293 cells cotransfected with vector constructs expressing (**a3**) GFP-Slc6a8a and Plum-Slc6a8b or (**b3**) Plum-Slc6a6 and GFP-Slc6a8a together with the respective profile of fluorescence intensities (ordinate) recorded at specific locations (abscissa) across the cell following the path as indicated by the dotted arrow in the panels. Nuclei were stained with Hoechst 33342 dye

The expected cellular localization of rainbow trout Slc6a8 proteins in the cell membrane was verified using labelled proteins. We tagged Slc6a8a with the green fluorescent protein GFP (GFP-slc6a8a) and Slc6a8b with the purple fluorescent protein Plum (Plum-Slc6a8b) and expressed them in human HEK cells (Fig. 5). Both variants, Slc6a8a (Fig. 5 (a1, b2)) and Slc6a8b (Fig. 5 (a2)), share the same subcellular localization in the cell membrane, similar as the taurine transporter Slc6a6 (Fig. 5 (b1)) and as the human ortholog (Rudnick et al. 2014). The localization of the rainbow trout creatine transporter in the cell membrane is confirmed by the highest accumulation of fluorescence intensities (Fig. 5 (a3, b3)) at the outer edge of the cell. The line scan analysis indicates overlapping membrane localization of GFP-Slc6a8a and Plum-Slc6a8b; however, sub-diffraction micro-domain differences cannot be resolved with the applied imaging approach.

### Detection of creatine in salmonid muscle extracts and analysis of Slc6a8 transport function using HPLC analysis

**Fig. 6:**
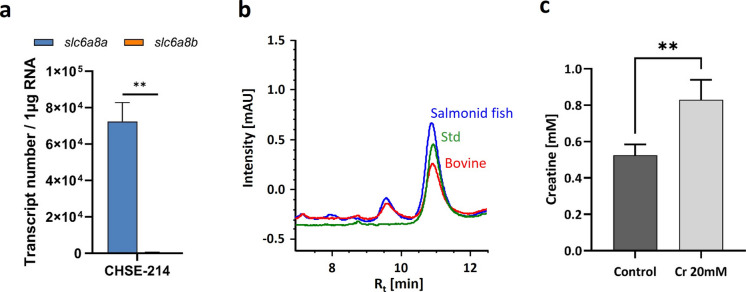
Creatine transport in salmonid fish cells. **(a**) Bars represent the transcript level of rainbow trout *slc6a8* gene variants in the salmonid cell line CHSE-214. Copy numbers were normalised to three reference genes; error bars represent SEM. Asterisks indicate statistical significance: **p < 0.01. (**b)** Chromatogram of creatine extracted from muscle samples of salmonid fish (blue line) and bovine (red line). A standard of 2 mM creatine (green line) was used as external control. **(c)** Determination of creatine concentration in salmonid cell line CHSE-214. Bars represent the averaged (*n* = 3, ±SEM) creatine concentrations in CHSE-214 cells pre-incubated with 20 mM creatine (light grey bar) compared to the untreated control (dark grey bar)

Creatine is transported into the cell from the bloodstream via the creatine symporter CT1 (Christie 2007). In order to evaluate the specific functionality of the rainbow trout creatine transporters *in vitro*, we first determined the basic modulation of *slc6a8a* and *slc6a8b* in the salmonid cell line CHSE-214. We deliberately selected the CHSE-214 cell line because it represents a physiologically relevant teleost model system (Thangaraj et al. 2021), allowing the analysis of creatine accumulation in a fish cellular context. *Slc6a8a* showed a high transcript level whereas the mRNA concentration of *slc6a8b* was minimal, with only 449 transcripts per 1 µg RNA (Fig. 6a). Secondly, we transfected the CHSE-214 cells with vector constructs expressing *slc6a8a* and *slc6a8b* to measure alterations in the creatine concentration in the cells after incubation with 20 mM creatine. However, the intrinsic expression of creatine transporter *slc6a8a* seems to mask a possible effect of additional expression of the two *slc6a8*-expression vectors. Vector-DNA concentrations of 30 ng, 300 ng and 3000 ng showed no effect on the cellular creatine uptake (data not shown). Therefore, we performed HPLC analysis to indirectly determine the creatine transport by Slc6a8 into the salmonid cell line. This HPLC experiment was intended as a supportive biochemical analysis rather than as a comprehensive characterization of creatine occurrence in fish muscle, which has been documented previously (Borchel et al. 2019; Hunter 1929). We compared the amount of creatine uptake into the cells with and without pre-incubation with 20 mM creatine. For this purpose, creatine was initially detected in muscle lysates by comparison with a bovine reference sample. The detected retention time (*R*_t_) of 11.1 min matched that of the bovine muscle reference sample (Fig. 6b). The pre-incubation of the CHSE-214 cells with creatine increased its concentration in the lysate significantly (*p* = 0.003) from 0.52 to 0.83 mM, indicating its carrier-dependent transport across the cell membrane (Fig. 6c). The significant accumulation of creatine following exogenous application demonstrates creatine uptake in the cell model and is consistent with transporter-mediated creatine transport across the cell membrane. Due to the high endogenous expression of *slc6a8a* in CHSE-214 cells, the assay reflects the overall creatine accumulation in the cell model but does not allow unequivocal conclusions regarding the individual contributions of the paralogs *slc6a8a* and *slc6a8b* to cellular creatine transport. Future studies investigating the transport activity and regulatory mechanisms of the two creatine transporter genes will contribute to a more detailed characterisation of their distinct functional roles within the creatine metabolism of rainbow trout. In this context, *Xenopus laevis* oocytes constitute a well-established heterologous expression system for detailed transporter kinetic and electrophysiological analyses due to their low endogenous transporter background (Bhatt et al. 2022). Together with gene knock-out approaches, *Xenopus laevis* oocytes may provide a suitable environment for future paralog-specific characterization of Slc6a8a and Slc6a8b transport properties.

In summary, we characterised two gene variants of the creatine transporter *slc6a8* in rainbow trout at both the structural and functional levels. Our analyses confirm their classification as membrane transporters and suggest their subfunctionalisation across tissues and in a salmonid cell model. We also demonstrated divergent retention of the *slc6a8* duplication in teleost fish compared to higher vertebrates. Moreover, the variant-specific expression patterns, together with the distribution of key creatine metabolism enzymes, support the existence of an evolutionarily distinct creatine metabolism in fish relative to mammals. Future research should aim to deepen the functional understanding of the *slc6a8* gene variants, for instance by testing the hypothesis that they may preferentially function as importer and exporter in distinct tissues, under specific physiological conditions, in order to uncover their specific properties and further clarify their respective role in the creatine metabolism of teleost fish.

## Supplementary information

Below is the link to the electronic supplementary material.

ESM 1 **Supplementary file: Fig.S1 Comparative alignment of predicted Slc6a8a (XP_036803444) and Slc6a8b (XP_021422671) protein sequences from rainbow trout.** The symbols below the sequences indicate identical (*), strongly similar (:) and weakly similar (.) amino acid residues. Conserved sections of amino acids are highlighted in blue and framed by blue boxes. The lines above and below the sequences mark intracellular (light grey) and transmembrane domains (apricot). Predicted helical structures are red shadowed and named above (Slc6a8a: α1–15) and below (Slc6a8b: α1–17) the sequences. (PDF 1.55 MB); Table S1: Gene-specific primers used in this study; Table S2: Accession numbers of sequences used for the phylogenetic tree.

ESM 2 (XLSX 245 KB)

## Data Availability

Most data generated and analysed during the current study are included in this published article and its Supplementary Information files. Previously published sequencing datasets re-analysed in this study are available from the original publications and their respective public repositories. Additional raw data are available from the corresponding author upon reasonable request.
