# Supplementary material for: Fuelled by creatine: exploring two copies of the creatine transporter SLC6A8 gene in rainbow trout
Source: Fish Physiol Biochem. 2026 Jun 13;52(3):96. doi: 10.1007/s10695-026-01723-y (PMC13264567; doi:10.1007/s10695-026-01723-y)
Supplement: Supplementary file 1 — Supplementary file: Fig.S1 Comparative alignment of predicted Slc6a8a (XP_036803444) and Slc6a8b (XP_021422671) protein sequences from rainbow trout. The symbols below the sequences indicate identical (*), strongly similar (:) and weakly similar (.) amino acid residues. Conserved sections of amino acids are highlighted in blue and framed by blue boxes. The lines above and below the sequences mark intracellular (light grey) and transmembrane domains (apricot). Predicted helical structures are red shadowed and named above (Slc6a8a: α1–15) and below (Slc6a8b: α1–17) the sequences. (PDF 1.55 MB); Table S1: Gene-specific primers used in this study; Table S2: Accession numbers of sequences used for the phylogenetic tree. [file 10695_2026_1723_MOESM1_ESM.pdf]

Fig.S1

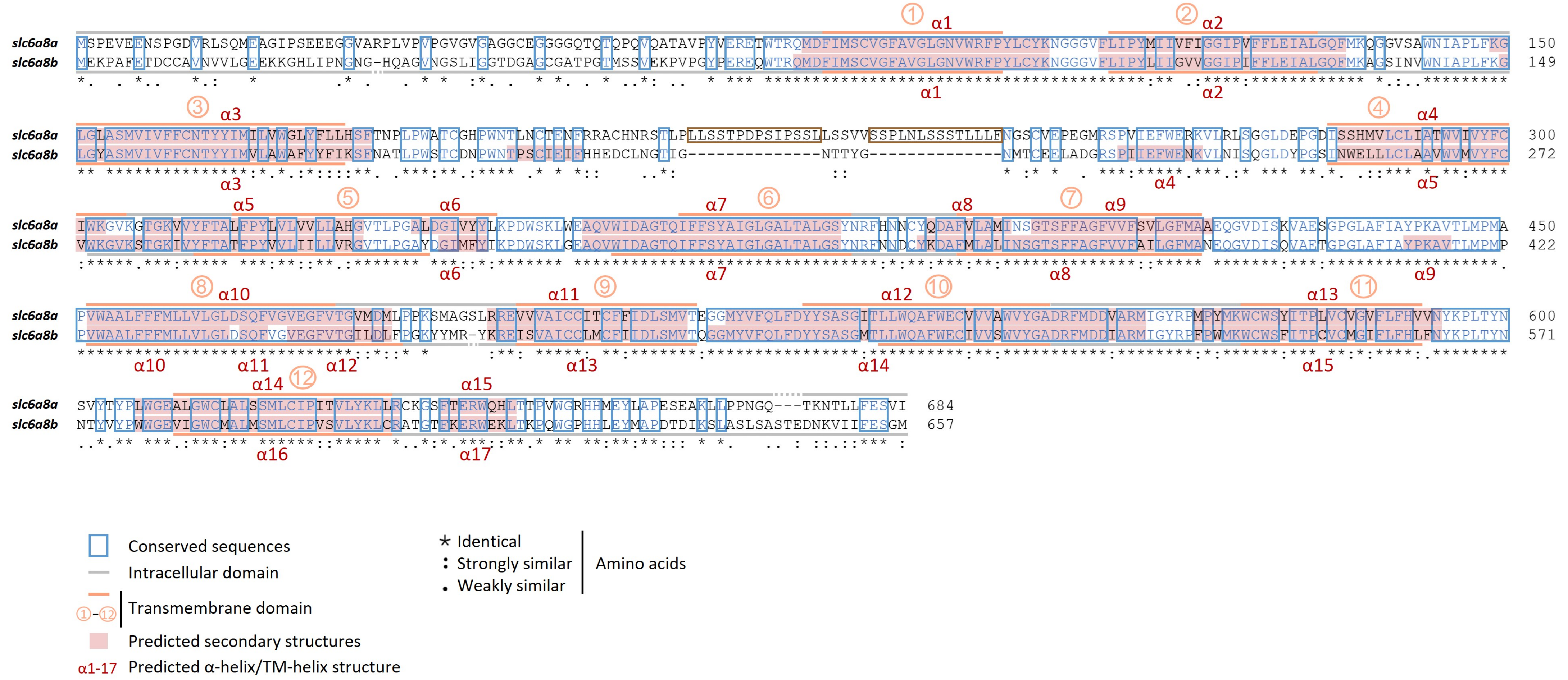
slc6a8a MSPEVEENSPGDVRLSQMEAGIPSEEEGGVARPLVPVPGVGVGAGGCEGGGGQTQTQPQVQATAVPYVERETWTRQMDFIMSCVGFAVGLGNVWRFPYLCYKNGGGVFLIPYMIIVFIGGIPVFFLEIALGQFMKQGGVSAWNIAPLFKG 150
slc6a8b MEKPAFETDCCAVNVVLGEEKKGHLIPNGNG-HQAGVNGSLIGGTDGAGCGATPGTMSSVEKPVPGYPEREQWTRQMDFIMSCVGFAVGLGNVWRFPYLCYKNGGGVFLIPYLIIGVVGGIPIFFLEIALGQFMKAGSINVWNIAPLFKG 149
slc6a8a LGLASMVIVFFCNTYYIMILVWGLYFLLHSFTNPLPWATCGHPWNTLNCTENFRRACHNRSTLPLLSSTPDPSIPSSLLSSVVSSPLNLSSSTLLLFNGSCVEPEGMRSPVIEFWERKVLRLSGGLDEPGDISSHMVLCLIATWVIVYFC 300
slc6a8b LGYASMVIVFFCNTYYIMVLAWAFYYFIKSFNATLPWSTCDNPWNTPSCIEIFHHEDCLNGTIG--------------NTTYG-------------NMTCEELADGRSPIIEFWENKVLNISQGLDYPGSINWELLCLAAVWVMVYFC 272
slc6a8a IWKGVKGTGKVVYFTALFPYLVLVVLLAHGVTLPGALDGIVYYLKPDWSKLWEAQVWIDAGTQIFFSYAIGLGALTALGSYNRFHNNCYQDAFVLAMINSGTSFFAGFVVFSVLGFMAAEQGVDISKVAESGPGLAFIAYPKAVTLMPMA 450
slc6a8b VWKGVKSTGKIVYFTATFPYVVLIILLVRGVTLPGAYDGIMFYIKPDWSKLGEAQVWIDAGTQIFFSYAIGLGALTALGSYNRFNNDCYKDAFMLALINSGTSFFAGFVVFAILGFMANEQGVDISQVAETGPGLAFIAYPKAVTLMPMP 422
slc6a8a PVWAALFFFMLLVLGLDSQFVGVEGFVTGVMDMLPPKSMAGSLRREVVVAICCITCFFIDLSMVTEGGMYVFQLFDYYSASGITLLWQAFWECVVVAWVYGADRFMDDVARMIGYRPMPYMKWCWSYITPLVCVGVFLFHVVNYKPLTYN 600
slc6a8b PVWAALFFFMLLVLGLDSQFVGVEGFVTGILDLFPGKYYMR-YKREISVAICCLMCFIIDLSMVTQGGMYVFQLFDYYSASGMTLLWQAFWECIVVSWVYGADRFMDDIARMIGYRPFPWMKWCWSFITPCVCMGIFLFHLFNYKPLTYN 571
slc6a8a SVYTYPLWGEALGWCLALSSMLCIPITVLYKLLRCKGSFTERWQHLTTPVWGRHHMEYLAPESEAKLLPPNGQ---TKNTLLFESVI 684
slc6a8b NTYVYPWWGEVIGWCMALMSMLCIPVSVLYKLCRATGTFKERWEKLTKPQWGPHHLEYMAPDTDIKSLASLSASTEDNKVIIFESGM 657
α1 α2 α3 α4 α5 α6 α7 α8 α9 α10 α11 α12 α13 α14 α15 α16 α17
Conserved sequences
Intracellular domain
Transmembrane domain
Predicted secondary structures
α1-17 Predicted α-helix/TM-helix structure
* Identical
: Strongly similar
. Weakly similar
Amino acids

Table S1 Gene-specific primers used in this study

Primer Name	NCBI Accession Number	Sense Primer (5′-3′)	Antisense Primer (5′-3′)	Fragment Length [bp]
Construction of expression constructs:				
slc6a6	XM_021615500	CCCAAGCTTCTGATATGGCACAGAAAGAGAAGCTACAA	CCCAGCGCTCATCATTGTTTCTACAATGGTGTGGGT	1901
slc6a8a	XM_036947549	CCCAAGCTTATGTCGCCAGAAGTGGAAGAGA	CCGGAATTCGATGACACTCTCAAACAGCAAAGTG	2073
slc6a8b	XM_021566996	CCCCAATTGCTGATATGGAGAAACCTGCATTCG	CCCAGCGCTCATACCACTTTCAAAGATGATAACC	1994
Quantitative PCR analysis:				
slc6a8a	XM_036947549	TGGGTCTATGGTGCAGATCGTT	TAGGTCAGGGGCTTGTAGTTCA	155
slc6a8b	XM_021566996	GTTACACAGGGAGGGATGTATG	CATCCAAGGAAAAGGCCGGTAA	174

Table S2: Accession numbers of sequences used for the phylogenetic tree.

Gene Symbol	Full Gene Name	Organism	Accession Number Protein
SLC4A1	**solute carrier family 4 member 1**	*Homo sapiens*	NP_000333
		Gallus gallus	XP_414303
		Xenopus tropicalis	XP_002941943
SLC6A1	**solute carrier family 6 member 1**	*Homo sapiens*	NP_001335179
		Oncorhynchus mykiss	XP_036806854
		Salmo salar	XP_045547929
slc6a1a	solute carrier family 6 member 1a	*Takifugu rubripes*	XP_029689431
		Danio rerio	NP_001038752
slc6a1b	solute carrier family 6 member 1b	*Salmo salar*	XP_014023838
		Takifugu rubripes	XP_003973460
		Danio rerio	NP_001007363
SLC6A2	**solute carrier family 6 member 2**	*Homo sapiens*	NP_001034
		Gallus gallus	NP_990047
		Danio rerio	XP_694138
		Xenopus tropicalis	XP_017948962
		Takifugu rubripes	XP_011607998
		Oncorhynchus mykiss	XP_021442268
SLC6A3	**solute carrier family 6 member 3**	*Homo sapiens*	NP_001035
		Danio rerio	NP_571830
		Xenopus tropicalis	XP_004915389
		Takifugu rubripes	XP_029694770
		Salmo salar	XP_045575284
		Oncorhynchus mykiss	XP_036791048
SLC6A4	**solute carrier family 6 member 4**	*Homo sapiens*	NP_001036

		Gallus gallus	NP_998737
		Salmo salar	XP_045559562
		Oncorhynchus mykiss	XP_036817964
		Danio rerio	NP_001035061
slc6a4a	solute carrier family 6 member 4a	*Xenopus tropicalis*	XP_017946918
		Takifugu rubripes	XP_003968295
		Danio rerio	NP_001170930
slc6a4b	solute carrier family 6 member 4b	*Xenopus tropicalis*	XP_012825214
		Takifugu rubripes	XP_003975030
		Homo sapiens	NP_004202
		Gallus gallus	XP_025007020
		Danio rerio	NP_001009557
SLC6A5	**solute carrier family 6 member 5**	*Xenopus tropicalis*	XP_031756248
		Takifugu rubripes	XP_003969823
		Salmo salar	XP_013981709
		Oncorhynchus mykiss	XP_021432569
		Homo sapiens	NP_003034
		Gallus gallus	XP_046755869
SLC6A6	**solute carrier family 6 member 6**	*Xenopus tropicalis*	XP_017949178
		Takifugu rubripes	XP_003963395
		Oncorhynchus mykiss	XP_021471175
slc6a6a	solute carrier family 6 member 6a	*Danio rerio*	NP_001119858
slc6a6b	solute carrier family 6 member 6b	*Danio rerio*	NP_001032750
		Salmo salar	NP_001133272
		Homo sapiens	NP_055043
		Gallus gallus	XP_414596
SLC6A7	**solute carrier family 6 member 7**	*Danio rerio*	NP_001074049
		Xenopus tropicalis	XP_002940131

		Takifugu rubripes	XP_003971273
		Salmo salar	NP_001167328
		Oncorhynchus mykiss	XP_021473908
SLC6A8	**solute carrier family 6 member 8**		
		Gallus gallus	XP_015148815
		Xenopus tropicalis	XP_012815966
Slc6a8a	solute carrier family 6 member 8a	*Danio rerio*	XP_001922963
		Takifugu rubripes	XP_003963342
		Salmo salar	XP_045548961
		Oncorhynchus mykiss	XP_036803444
		Homo sapiens	NP_005620
		Xenopus tropicalis	XP_002935539
SLC6A8b	solute carrier family 6 member 8b	*Takifugu rubripes*	XP_029690104
		Danio rerio	NP_001314797
		Oncorhynchus mykiss	XP_021422671
		Salmo salar	XP_013990051
		Homo sapiens	NP_964012
		Gallus gallus	NP_001026450
SLC6A9	**solute carrier family 6 member 9**	*Danio rerio*	NP_001025244
		Xenopus tropicalis	XP_004914027
		Takifugu rubripes	XP_011615027
		Oncorhynchus mykiss	XP_021468932
slc6a9l	solute carrier family 6 member 9 like	*Salmo salar*	XP_013995553
		Homo sapiens	NP_055044
SLC6A11	**solute carrier family 6 member 11**	*Gallus gallus*	XP_414302
		Xenopus tropicalis	XP_031757159
slc6a11a	solute carrier family 6 member 11a	*Danio rerio*	NP_001091857
		Takifugu rubripes	XP_011612184

		Salmo salar	XP_013987847
		Oncorhynchus mykiss	XP_021424558
		Danio rerio	XP_017213744
slc6a11b	solute carrier family 6 member 11b	*Salmo salar*	XP_014023836
		Takifugu rubripes	XP_003973461
		Oncorhynchus mykiss	XP_036840174
		Homo sapiens	NP_003035
SLC6A12	**solute carrier family 6 member 12**	*Gallus gallus*	XP_416378
		Xenopus tropicalis	XP_031754510
		Homo sapiens	NP_057699
		Gallus gallus	XP_004937983
SLC6A13	**solute carrier family 6 member 13**	*Danio rerio*	XP_002667408
		Xenopus tropicalis	NP_001072913
		Takifugu rubripes	XP_029698075
		Oncorhynchus mykiss	XP_036808456
slc6a13l	solute carrier family 6 member 13 like	*Salmo salar*	XP_014025348
		Homo sapiens	NP_009162
		Gallus gallus	XP_003641183
SLC6A14	**solute carrier family 6 member 14**	*Danio rerio*	NP_001313379
		Salmo salar	XP_014022987
		Oncorhynchus mykiss	XP_021466038
		Takifugu rubripes	XP_029683122
slc6a14	solute carrier family 6 member 14 gene 1	*Xenopus tropicalis*	XP_002939712
slc6a14.2	solute carrier family 6 member 14 gene 2	*Xenopus tropicalis*	XP_002939711
		Homo sapiens	NP_877499
SLC6A15	**solute carrier family 6 member 15**	*Gallus gallus*	XP_416124
		Danio rerio	NP_001232001
		Xenopus tropicalis	XP_004912967

		Takifugu rubripes	XP_003967814
slc6a15	solute carrier family 6 member 14 gene 1	*Salmo salar*	XP_013981187
slc6a15.2	solute carrier family 6 member 14 gene 2	*Salmo salar*	XP_014003294
slc6a15	solute carrier family 6 member 14 gene 1	*Oncorhynchus mykiss*	XP_021432461
slc6a15.2	solute carrier family 6 member 14 gene 2	*Oncorhynchus mykiss*	XP_021459775
SLC6A16	**solute carrier family 6 member 16**	*Homo sapiens*	NP_054756
		Xenopus tropicalis	XP_004916326
slc6a16a	solute carrier family 6 member 16a	*Danio rerio*	XP_021329024
slc6a16b	solute carrier family 6 member 16b	*Danio rerio*	NP_001103944
SLC6A17	**solute carrier family 6 member 17**	*Homo sapiens*	NP_001010898
		Gallus gallus	XP_046760170
		Danio rerio	NP_001243653
		Xenopus tropicalis	XP_002933031
slc6a17.1	solute carrier family 6 member 17 gene 1	*Oncorhynchus mykiss*	XP_036844818
slc6a17.2	solute carrier family 6 member 17 gene 2	*Oncorhynchus mykiss*	XP_036803620
slc6a17.3	solute carrier family 6 member 17 gene 3	*Oncorhynchus mykiss*	XP_036806317
slc6a17.4p	solute carrier family 6 member 17 gene 4 partial	*Oncorhynchus mykiss*	XP_036839536
slc6a17.1	solute carrier family 6 member 17 gene 1	*Salmo salar*	XP_013989838
slc6a17.2	solute carrier family 6 member 17 gene 2	*Salmo salar*	XP_045552409
slc6a17.3	solute carrier family 6 member 17 gene 3	*Salmo salar*	XP_013988485
slc6a17	solute carrier family 6 member 17 gene 1	*Takifugu rubripes*	XP_029690120
slc6a17.2	solute carrier family 6 member 17 gene 2	*Takifugu rubripes*	XP_003973303
SLC6A18	**solute carrier family 6 member 18**	*Homo sapiens*	NP_872438
		Gallus gallus	XP_003640835
		Danio rerio	NP_001108369
		Takifugu rubripes	XP_003969014
		Oncorhynchus mykiss	XP_021417749
SLC6A19	**solute carrier family 6 member 19**	*Homo sapiens*	NP_001003841

		Gallus gallus	XP_419056
		Xenopus tropicalis	NP_001120585
		Takifugu rubripes	XP_003969076
		Salmo salar	NP_001135287
		Oncorhynchus mykiss	XP_021428466
slc6a19a.1	solute carrier family 6 member 19a gene 1	*Danio rerio*	NP_001091648
slc6a19a.2	solute carrier family 6 member 19a gene 2	*Danio rerio*	XP_002665492
slc6a19b	solute carrier family 6 member 19b	*Danio rerio*	NP_956030
		Homo sapiens	NP_064593
		Gallus gallus	XP_418798
SLC6A20	**solute carrier family 6 member 20**	*Xenopus tropicalis*	XP_002942571
		Takifugu rubripes	XP_029695008
		Salmo salar	XP_014056732
